# High sodium intake and fluid overhydration predict cardiac structural and functional impairments in chronic kidney disease

**DOI:** 10.3389/fnut.2024.1388591

**Published:** 2024-05-27

**Authors:** Suyan Duan, Yuchen Ma, Fang Lu, Chengning Zhang, Honglei Guo, Ming Zeng, Bin Sun, Yanggang Yuan, Changying Xing, Huijuan Mao, Bo Zhang

**Affiliations:** Department of Nephrology, the First Affiliated Hospital of Nanjing Medical University, Nanjing Medical University, Nanjing, China

**Keywords:** chronic kidney disease, total body water, extracellular water, daily salt intake, left ventricular hypertrophy, elevated left ventricular filling pressure

## Abstract

**Background:**

High sodium intake and fluid overhydration are common factors of and strongly associated with adverse outcomes in chronic kidney disease (CKD) patients. Yet, their effects on cardiac dysfunction remain unclear.

**Aims:**

The study aimed to explore the impact of salt and volume overload on cardiac alterations in non-dialysis CKD.

**Methods:**

In all, 409 patients with CKD stages 1–4 (G1–G4) were enrolled. Daily salt intake (DSI) was estimated by 24-h urinary sodium excretion. Volume status was evaluated by the ratio of extracellular water (ECW) to total body water (TBW) measured by body composition monitor. Recruited patients were categorized into four groups according to DSI (6 g/day) and median ECW/TBW (0.439). Echocardiographic and body composition parameters and clinical indicators were compared. Associations between echocardiographic findings and basic characteristics were performed by Spearman’s correlations. Univariate and multivariate binary logistic regression analysis were used to determine the associations between DSI and ECW/TBW in the study groups and the incidence of left ventricular hypertrophy (LVH) and elevated left ventricular filling pressure (ELVFP). In addition, the subgroup effects of DSI and ECW/TBW on cardiac abnormalities were estimated using Cox regression.

**Results:**

Of the enrolled patients with CKD, the median urinary protein was 0.94 (0.28–3.14) g/d and estimated glomerular filtration rate (eGFR) was 92.05 (IQR: 64.52–110.99) mL/min/1.73 m^2^. The distributions of CKD stages G1–G4 in the four groups was significantly different (*p* = 0.020). Furthermore, compared to group 1 (low DSI and low ECW/TBW), group 4 (high DSI and high ECW/TBW) showed a 2.396-fold (95%CI: 1.171–4.902; *p* = 0.017) excess risk of LVH and/or ELVFP incidence after adjusting for important CKD and cardiovascular disease risk factors. Moreover, combined with eGFR, DSI and ECW/TBW could identify patients with higher cardiac dysfunction risk estimates with an AUC of 0.704 (sensitivity: 75.2%, specificity: 61.0%). The specificity increased to 85.7% in those with nephrotic proteinuria (AUC = 0.713). The magnitude of these associations was consistent across subgroups analyses.

**Conclusion:**

The combination of high DSI (>6 g/d) and high ECW/TBW (>0.439) independently predicted a greater risk of LVH or ELVFP incidence in non-dialysis CKD patients. Moreover, the inclusion of eGFR and proteinuria improved the risk stratification ability of DSI and ECW/TBW in cardiac impairments in CKD.

## Graphical abstract

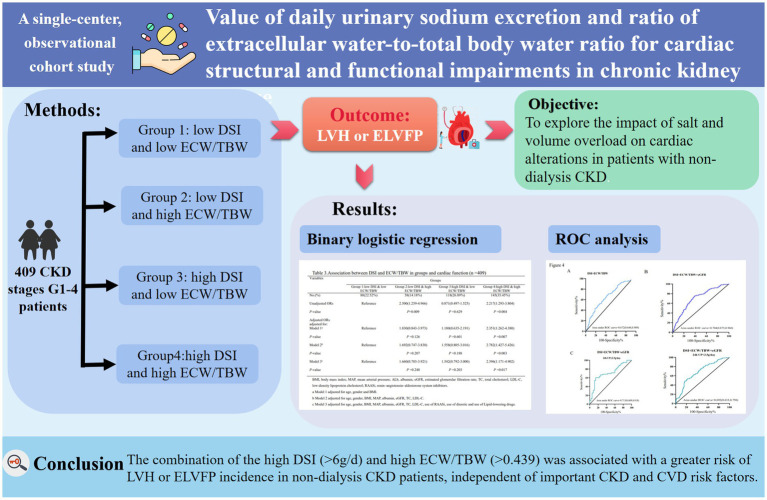

## Highlights

- High sodium intake and fluid overhydration are common in CKD patients and strongly associated with adverse outcomes, yet effects on cardiac dysfunction remain unclear.
- The combination of the high daily salt intake (DSI) (>6g/d) and high extracellular water (ECW)/total body water (TBW) (>0.439) is associated with a greater risk of of cardiac structural and functional impairments in non-dialysis CKD patients, independent of important CKD and CVD risk factors.
- Adding eGFR and proteinuria into consideration would improve the ability of DSI and ECW/TBW for risk stratification of cardiac impairments in CKD patients.
- Salt and volume overload may be important mechanisms contributing to the CVD morbidity and mortality observed in non-dialysis CKD. Timely modifying the salt and fluid status may be benefit for achieving better CVD outcomes and therefore decreasing the burden of mortality in CKD.

## 1 Introduction

Chronic kidney disease (CKD) has emerged as an increasingly important risk factor contributing to cardiovascular diseases (CVD), which is currently the leading cause of morbidity and mortality worldwide (1, 2). Numerous epidemiological studies have reported a strong association between deteriorating renal function and ultimate outcomes of CVD, and severe CV events accounted for almost 50% of all deaths in the population with kidney disease (2–4). According to the 2019 Global Burden of Disease study, 15–20% of the world’s population is affected by CKD, which is ranked the 12th leading risk factor affecting disability-adjusted life-years (5, 6). Thus, predicting CV risk and strategies for preventing the development of CV events in CKD would not only improve CKD outcomes but also give rise to significant amelioration of the heavy burden in public health and medical expenses.

Diastolic filling of the left ventricle (LV) is a highly complex process dependent on LV relaxation, LV compliance, and left atrial pressure, which is contributing to exercise capacity and quality of life (7). LV-filling pressure (LVFP), is recognized as a potential factor contributing to pulmonary congestion (8). Besides, the severity of CKD has been reported as the most independent predictors of elevated LVFP (ELVFP) (8). Among the echocardiographic parameters, the ratio of early diastolic mitral inflow velocity (E) to early diastolic mitral annular velocity (e’) has been proven to be a useful parameter for assessing LVFP (9–11). Recent European guidelines on heart failure (HF) proposed a resting average E/e’ > 9 as an objective criterion of ELVFP (12). Moreover, E/e’ > 9 showed an association with increased risk of CV and all-cause mortality (7). However, a huge body of evidence indicated that LVH was the most common structural abnormality associated with CKD patients, the prevalence of which ranged from 34 to 78%, with increasing values correlating with the worsening in renal function (13, 14). One of the main mechanisms involving LV hypertrophy (LVH) is a physiological response to pressure and volume overload (8, 15). Hence, exploring the risk factors of LVH and ELVFP not only provides a more in-depth understanding of the underlying mechanisms of the major complication but also highlights opportunities for potential therapeutic intervention with the aim to improve the CVD outcomes of CKD in clinical practice.

Salt and volume overload are key factors contributing to LVH, LV dilatation, and adverse CVD outcomes in CKD (14, 16). However, how to monitor salt and fluid balance in patients with non-dialysis CKD remains largely unclear in clinical practice. High salt intake as measured by 24-h urinary sodium excretion is associated with hypertension, increased urinary protein, occurrence of CVD, and kidney outcome in CKD (17–19). Volume overload is the consequence of excessive sodium loading by reduced glomerular filtration, and in turn contributes to hypertension in CKD patients (20). Moreover, volume overload has shown great promise as a potential modifiable risk factor of CKD progression and CVD (21). Hence, our primary aim was to analyze the effects of both daily salt intake (DSI) (by detecting 24-h urinary sodium excretion) and volume status as assessed by the ratio of extracellular water (ECW) to total body water (TBW) (measured using a body composition monitor) on cardiac abnormalities in patients with non-dialysis CKD. Additionally, we explored the specific group of patients with comorbid cardiac structural and functional impairments who may most benefit from water and sodium restriction.

## 2 Materials and methods

### 2.1 Subjects

**Figure 1:**
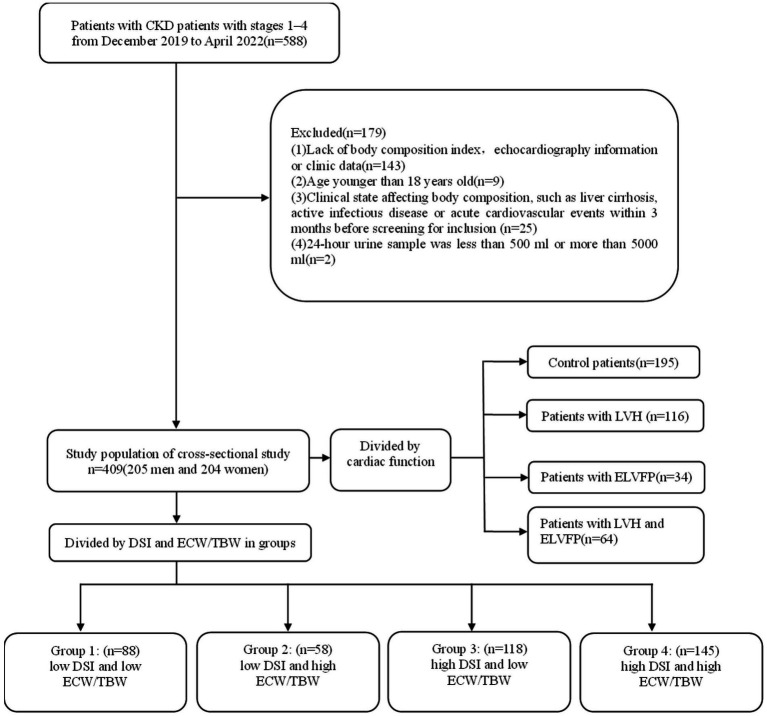
Flowchart of study participants. CKD, chronic kidney disease; TBW, total body water; ECW, extracellular water; DSI, daily salt intake; LVH, left ventricular hypertrophy; ELVFP, elevated left ventricular filling pressure.

This was a observational cohort study with patients recruited from the Department of Nephrology in the First Affiliated Hospital of Nanjing Medical University from December 2019 to April 2022. The inclusion criteria were as follows: (1) patients diagnosed with CKD, which was defined as abnormalities of kidney structure or function that persist for ≥3 months based on the Kidney Disease Improving Global Outcomes (KDIGO) Clinical Practice Guidelines (22); (2) patients cooperating with body composition analysis; (3) intact echocardiography data; and (4) estimated glomerular filtration rate (eGFR) > 15 mL/min/1.73 m^2^. The exclusion criteria were as follows: (1) lack of body composition index and echocardiography information or clinic data; (2) age < 18 years; (3) clinical state affecting body composition, such as liver cirrhosis, active infectious disease, or acute CV events within 3 months before screening for inclusion; and (4) a 24-h urine sample either <500 mL or > 5,000 mL. Eventually, 409 patients were eligible for the analysis (Figure 1). This study was approved by the Ethics Committee of the First Affiliated Hospital of Nanjing Medical University (No. 2023-SR-178) and conducted in accordance with the guidelines of the Declaration of Helsinki.

### 2.2 Clinical and laboratory parameters

The complete clinical and laboratory information of enrolled patients was collected during hospitalization from medical records and included age, sex, blood pressure, history of diabetes mellitus (DM), history of hypertension, body mass index (BMI), hemoglobin (Hb), albumin (ALb), total cholesterol (TC), triglyceride (TG), high-density lipoprotein cholesterol (HDL-C), low-density lipoprotein cholesterol (LDL-C), uremic acid (UA), serum creatine (Scr), eGFR, blood urea nitrogen (BUN), 24-h urinary protein excretion (24-h UP), and 24-h urinary sodium. All 24-h urine samples were collected from the day of admission to the following day. In addition, we also recorded the use of drugs such as renin–angiotensin–aldosterone system inhibitors (RAASi), diuretics, and lipid-lowering drugs.

Furthermore, DSI was estimated from 24-h urinary sodium excretion (19, 23, 24). The calculation was performed with the following formula: DSI (g/d) = urinary sodium concentration (mmol/L) × 24-h urine volume (L) × 0.05842 (g/mmol). Based on it, patients were categorized into two groups according to DSI with a cut-off level of 6 g/day, which is recommended on the basis of evidence-based clinical practice guidelines for CKD (19, 25–28).

### 2.3 Measurement of volume status

All patients were analyzed using the body composition monitor (Fresenius Medical Care, Bad Homburg, Germany), by a designated technician according to the manufacturer’s instructions. Hydration status including TBW, ECW, intracellular water (ICW), and overhydration (OH) was recorded (29, 30). ECW/TBW has been recognized as a notable indicator of adverse outcomes of a composite of volume overload and protein-energy wasting (31, 32). Accordingly, patients were then divided into two groups as per the median ECW/TBW.

### 2.4 Divisions

Four groups were divided according to both DSI and ECW/TBW as previously reported (19): Group 1, low DSI (<6 g/day) and low ECW/TBW (<median value 0.439); Group 2, low DSI (<6 g/day) and high ECW/TBW (≥median value 0.439); Group 3, high DSI (≥6 g/day) and low ECW/TBW (<median value 0.439); and Group 4, high DSI (≥6 g/day) and high ECW/TBW (≥median value 0.439).

### 2.5 Echocardiographic measurements

Echocardiographic data was acquired by an ultrasound machine (Vivid E9; GE Vingemed Ultrasound AS, Horten, Norway) with a 2.5-MHz transducer. All individuals were assessed for diastolic function measurement including mitral E wave velocity, mitral A wave velocity, mitral E:A ratio, septal or lateral mitral annular e’ velocity (e’ velocity), and E:e’ ratio. The value of E/e’ was helpful to diagnose the diastolic dysfunction of the left ventricle (15). E/e’ > 9 was associated with increased risk of cardiovascular and all-cause mortality and was also used as an indicator to define ELVFP (7, 10, 11, 15).

Cardiac chamber quantification was performed in accordance with the American Society of Echocardiography guidelines (33). The dimensions of the interventricular septal thickness (IVST), left ventricular end-diastolic dimension (LVDd), left ventricular posterior wall thickness (PWT), left atrial dimension (LAD), and left ventricular ejection fraction (LVEF) using the biplane Simpson’s method were measured. IVST and PWT were measured at end-diastole. Left ventricular hypertrophy (LVH) was defined as an LVMI>115 g/m^2^ in male and > 95 g/m^2^ in female patients (33).

The cardiac data was calcuated using the following formula: Left ventricular mass = 0.8 × 1.04 × [(IVST+LVDd+PWT)^3^–LVDd^3^] + 0.6 (g). Body surface area (BSA) = {([height (cm) × weight (kg)]/3600)^×½^)}m^2^. Left ventricular mass index (LVMI) = LVM/BSA (g/m^2^). Relative wall thickness (RWT) = 2 × PWT/LVDd.

### 2.6 Statistical analysis

Categorical variables were reported in percentage and continuous variables were calculated for mean ± SD or medians and interquartile ranges with or without normal distribution, respectively. Comparisons of categorical variables among the four groups were performed by chi-squared test. Mann–Whitney test and Kruskal–Wallis test were used to compare continuous variables as appropriate.

The correlations between echocardiographic parameters and clinical indicators were assessed using Spearman’s correlation analysis. Univariate and multivariate binary logistic regression analysis were performed to determine the associations between DSI and ECW/TBW in groups and cardiac dysfunction. Multiple covariables were adjusted. In the fully adjusted model, factors included but not limited to age, sex, BMI, and cardiovascular health indicators were used for adjustment. Subgroup analyses were performed for serum albumin, eGFR, BMI, 24-h urinary protein, and history of hypertension and DM in the association between DSI, ECW/TBW, and cardiac impairments.

A *p* value of less than 0.05 was considered to indicate statistically significant differences between groups. And the analyses were performed using SPSS version 27.0 (IBM Corporation, Armonk, NY, USA).

## 3 Results

### 3.1 Basic characteristics

**Figure 2:**
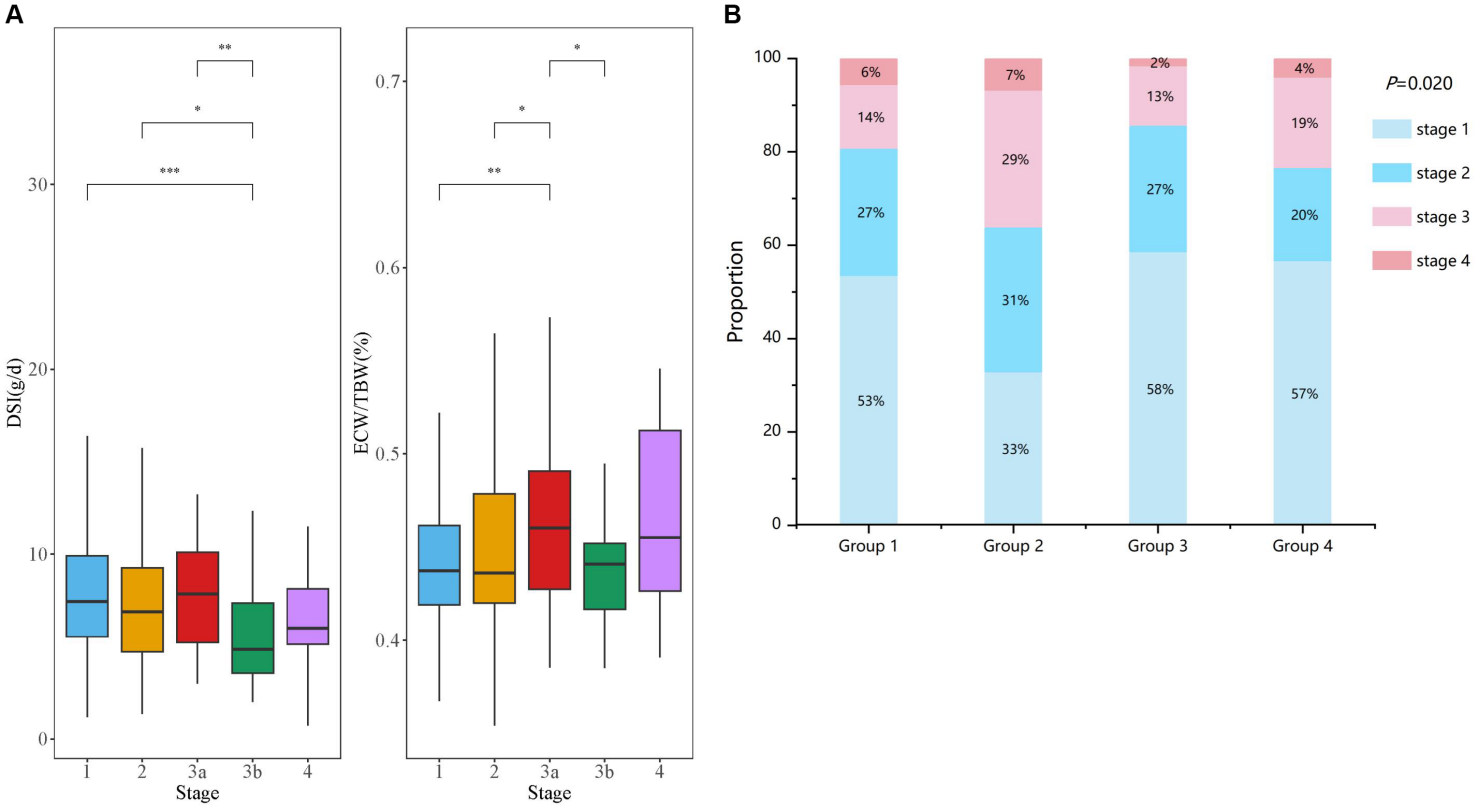
Comparison of DSI and ECW/TBW in CKD patients with different stages. **(A)** Comparisons of the levels of DSI and ECW/TBW among all enrolled patients are shown at stages G1–G4 of CKD. **(B)** The four groups were divided according to both DSI and ECW/TBW. Proportion of CKD stages G1–G4 are displayed in groups 1–4 **(B)**.

A total of 409 patients (205 men and 204 women) with stages G1-G4 CKD (53.05, 25.18, 17.60, and 4.16% at different stages respectively) were recruited (Figure 1; Table 1). The median age of patients was 45.00 (IQR: 32.00–56.00) years, with a mean BMI of 24.59 ± 3.74 kg/m^2^. The median urinary protein was 0.94 (0.28–3.14) g/d and eGFR was 92.05 (IQR: 64.52–110.99) mL/min/1.73 m^2^. In terms of echocardiographic parameters, the median LVMI, E/e’, and LVEF were 97.76 (82.21–115.38) g/m^2^, 7.20 (6.00–8.80), and 64.00 (62.40–65.80) %, respectively. The DSI estimated from 24-h urinary sodium excretion was 7.13 (IQR: 5.04–9.65) g, and the median ECW/TBW was 0.439 (IQR: 0.421–0.468). DSI was at the lowest level in stage G3b, followed by CKD G4 (G3b vs. G1: *p* < 0.001; G3b vs. G2: *p* < 0.05; G3b vs. G3a: *p* < 0.01) (Figure 2A). Moreover, there was a significant increase in ECW/TBW at stage G3a (G3a vs. G1: *p* < 0.01; G3a vs. G2: *p* < 0.05; G3a vs. G3b: *p* < 0.05) (Figure 2A).

**Table 1:** Baseline characteristics stratified by DSI and ECW/TBW categories (*n* = 409).

Variables	Total (*n* = 409)	DSI				*p* value
		Low		High		
		ECW/TBW				
		Low (group 1:*n* = 88)	High (group 2:*n* = 58)	Low (group 3:*n* = 118)	High (group 4:*n* = 145)	
Age (years)	45.00 (32.00–56.00)	35.50 (29.00–50.00)	53.50 (39.75–66.00)	38.00 (30.75–50.00)	51.00 (40.50–59.00)	*p* < 0.001
Gender (male/female)	205/204	42/46	21/37	77/41	65/80	*p* = 0.043
**Clinical parameter**						
BMI(kg/m^2^)	24.59 ± 3.74	23.12 ± 3.02	24.48 ± 3.20	24.25 ± 3.98	25.80 ± 3.77	*p* < 0.001
MAP(mmHg)	98.67 (88.67–106.83)	97.33 (87.08–104.00)	100.50 (90.00–106.67)	95.33 (86.92–106.25)	100.67 (90.83–109.00)	*p* = 0.020
**ECHO**						
LAD (mm)	32.00 (30.00–36.00)	31.00 (28.00–32.75)	32.50 (28.75–37.00)	32.00 (30.00–35.00)	34.00 (31.50–38.00)	*p* < 0.001
LVDd (mm)	46.00 (43.00–48.00)	44.50 (42.00–47.00)	45.00 (42.00–48.00)	46.50 (44.00–49.00)	47.00 (44.00–49.00)	*p* < 0.001
LVMI (g/m^2^)	97.76 (82.21–115.38)	94.63 (80.54–111.34)	104.63 (81.91–120.91)	93.28 (80.88–106.93)	101.06 (86.45–121.81)	*p* = 0.021
E/e’	7.20 (6.00–8.80)	6.50 (5.60–7.77)	7.95 (6.48–9.33)	6.70 (5.90–8.00)	7.80 (6.55–9.65)	*p* < 0.001
LVEF (%)	64.00 (62.40–65.80)	64.40 (63.08–66.03)	64.00 (62.10–66.50)	64.40 (63.00–65.90)	63.70 (62.10–65.40)	*p* = 0.076
**Bioelectrical impedance**						
OH (L)	0.20(−0.60–1.70)	−0.50(−1.08–0.10)	0.90 (0.10–5.40)	−0.45(−1.20–0.20)	1.40 (0.50–3.60)	*p* < 0.001
TBW (L)	35.30 (30.00–41.95)	34.20 (30.00–39.88)	30.95 (26.55–41.28)	37.70 (33.15–44.20)	35.30 (30.55–41.40)	*p* < 0.001
ECW (L)	15.60 (13.35–18.55)	14.10 (12.60–16.58)	14.15 (12.30–20.05)	15.70 (13.90–18.53)	17.40 (14.40–19.65)	*p* < 0.001
ICW (L)	19.80 (16.40–23.50)	20.05 (17.20–23.58)	15.65 (13.98–20.20)	21.90 (18.98–26.18)	18.70 (16.00–21.95)	*p* < 0.001
ECW/ICW	0.78 (0.72–0.88)	0.73 (0.68–0.76)	0.93 (0.84–1.00)	0.73 (0.69–0.75)	0.88 (0.83–0.97)	*p* < 0.001
OH/ECW (×10^−2^)	1.32(−4.29–9.46)	−3.08(−7.49–0.81)	6.94 (0.85–26.47)	−3.03(−7.29–1.24)	9.03 (2.93–19.94)	*p* < 0.001
ECW/TBW	0.439 (0.421–0.468)	0.421 (0.405–0.432)	0.478 (0.454–0.502)	0.421 (0.407–0.430)	0.466 (0.454–0.493)	*p* < 0.001
**Laboratory parameter**						
Hb (g/L)	129.88 ± 21.03	135.43 ± 18.82	124.16 ± 23.42	137.68 ± 19.02	122.46 ± 19.76	*p* < 0.001
ALb (g/L)	36.70 (28.40–40.05)	38.60 (36.35–41.60)	29.65 (21.73–37.83)	39.60 (36.00–42.30)	30.70 (22.35–36.95)	*p* < 0.001
TC (mmol/L)	4.86 (4.16–6.03)	4.67 (3.98–5.58)	5.20 (4.02–7.14)	4.65 (3.93–5.38)	5.35 (4.47–6.50)	*p* < 0.001
TG (mmol/L)	1.50 (1.01–2.23)	1.23 (0.90–1.83)	1.93 (1.26–2.70)	1.38 (0.90–2.00)	1.65 (1.11–2.39)	*p* < 0.001
HDL-C (mmol/L)	1.14 (0.96–1.41)	1.13 (0.96–1.40)	1.22 (0.99–1.57)	1.08 (0.93–1.34)	1.18 (0.99–1.38)	*p* = 0.051
LDL-C (mmol/L)	3.03 (2.45–3.73)	2.91 (2.30–3.51)	3.05 (2.32–4.09)	2.86 (2.36–3.46)	3.31 (2.74–4.11)	*p* < 0.001
eGFR (ml/min/1.73 m2)	92.05 (64.52–110.99)	91.47 (70.48–112.13)	73.15 (48.83–101.04)	97.08 (77.79–114.61)	95.62 (60.98–110.90)	*p* = 0.003
CKD stage (1/2/3a/3b/4)	217/103/42/30/17	47/24/2/10/5	19/18/11/6/4	69/32/11/4/2	82/29/18/10/6	*p* = 0.002
Scr (umol/L)	79.10 (60.50–105.30)	81.35 (66.33–106.15)	88.85 (61.53–117.65)	79.00 (63.23–100.83)	74.40 (57.50–97.50)	*p* = 0.072
24 h UP (g/d)	0.94 (0.28–3.14)	0.48 (0.17–1.06)	1.95 (0.69–7.35)	0.39 (0.16–1.26)	2.43 (0.64–5.93)	*p* < 0.001
24 h urinary sodium (mmol/d)	122.00 (86.30–165.15)	77.25 (63.13–88.43)	76.30 (59.08–87.90)	145.35 (120.53–191.40)	154.40 (127.35–194.60)	*p* < 0.001
DSI (g/d)	7.13 (5.04–9.65)	4.51 (3.69–5.17)	4.46 (3.45–5.14)	8.49 (7.04–11.18)	9.02 (7.44–11.37)	*p* < 0.001
**Medications**						
RAASi (%)	218 (53.30)	46 (52.27)	35 (60.34)	61 (51.69)	76 (52.41)	*p* = 0.715
Diuretic (%)	21 (5.13)	1 (1.14)	11 (18.97)	1 (0.84)	8 (5.51)	*p* < 0.001
Lipid-lowering drugs (%)	67 (16.38)	10 (11.36)	15 (25.86)	15 (12.71)	27 (18.62)	*p* = 0.068

BMI, body mass index; MAP, mean arterial pressure; ECHO, Echocardiography; LAD, left atrial dimension; LVDd, left ventricular end-diastolic dimension; LVMI, left ventricular mass index; LVEF, left ventricular ejection fraction; OH, overhydration; TBW, total body water; ECW, extracellular water; ICW, intracellular water; Hb, hemoglobin; ALb, albumin; TC, total cholesterol; TG, triglyceride; HDL-C, high-density lipoprotein cholesterol; LDL-C, low-density lipoprotein cholesterol; eGFR, estimated glomerular filtration rate; Scr, serum creatine; UP, urinary protein; DSI, daily salt intake; RAASi, renin–angiotensin–aldosterone system inhibitors.

Data were presented as the mean ± SD, the median with interquartile range or counts, and percentages. A two-tailed *p* < 0.05 was considered statistically significant.

Next, based on DSI estimated from 24-h urinary sodium excretion and ECW/TBW, all enrolled patients were categorized into four groups (Table 1). Patients with both low level of DSI and low level of ECW/TBW were in group 1 and those with both high level of DSI and high level of ECW/TBW were in group 4. Patients with high ECW/TBW (groups 2 and 4) were significantly older, female, and had a higher level of BMI and MAP than those with ECW/TBW below the median value (groups 1 and 3) regardless of the DSI level (*p* < 0.05 for all four groups). Echocardiographic parameters including LAD, LVDd, LVMI, and E/e’ were significantly different among the four groups (all *p* < 0.05). Patients in group 2, with low DSI and high ECW/TBW, had the highest values of LVMI and E/e’, which was 104.63 (81.91–120.91) g/m^2^ and 7.95 (6.48–9.33), respectively. With respect to bioelectrical impedance data, OH, ECW, and OH/ECW were highest in group 4 and lowest in group 1 (*p* < 0.05 among four groups). Meanwhile, TBW and ICW were highest in group 3 and lowest in group 2 (*p* < 0.05 among four groups). The levels of Hb, Alb, and LDL-C were higher; those of TC and TG were lower in groups 1 and 3 than in groups 2 and 4; furthermore, groups 1 and 3 required less frequent use of diuretics than groups 2 and 4(*p* < 0.05 among four groups). Kidney function was worst in group 2, with the lowest level of eGFR (73.15 [48.83–101.04] ml/min/1.73 m^2^) and highest level of Scr (88.85 [61.53–117.65] umol/L) (*p* < 0.05 among four groups). Besides, patients in group 4 had the highest level of 24-h UP (2.43 [0.64–5.93] g/d), followed by those in group 2 (1.95 [0.69–7.35] g/d). As displayed in Figure 2B, the distributions of CKD stages G1–G4 in the four groups was significantly different (*p* = 0.020). Patients in group 3 had the highest proportion of CKD stages G1 and G2 (85%) along with the lowest proportion of G3 and G4 (15%). Additionally, the highest ratio of stages G3 and G4 (36%) were observed in group 2.

### 3.2 Comparisons of baseline parameters according to echocardiographic indicators

According to cardiac function, 195 patients were categorized as controls, 116 patients had LVH, 34 patients had ELVFP, and 64 patients had LVH + ELVFP (Table 2). Compared to control patients, those in other three groups were significantly older (vs. control patients, *p* < 0.01 for each). BMI was significantly different among all four groups (*p* < 0.001), and the LVH group had the lowest BMI (*p* < 0.01). There were significant differences in echocardiographic parameters including LAD, LVDd, LVMI, and E/e’ among the four groups (all *p* < 0.001). All bioelectrical impedance parameters including OH, TBW, ECW, ICW, ECW/ICW, OH/ECW, and ECW/TBW showed statistically significant differences. More specifically, the ELVFP group had the highest level of OH, TBW, ECW, and OH/ECW, and the LVH + ELVFP group had the highest level of ECW/ICW and ECW/TBW (vs. control patients, *p* < 0.01). Meanwhile, the LVH group had the lowest level of TBW, ECW, and ICW (vs. control patients, *p* < 0.01). With respect to laboratory parameters, the LVH group had significantly decreased levels of Hb and Alb, but increased levels of HDL-C; whereas, the ELVFP group had significant lower Alb levels (vs. control patients, *p* < 0.01; *p* < 0.05 among four groups). Kidney function was significantly different among all four groups, and the LVH + ELVFP group had the lowest level of eGFR, followed by the ELVFP group (*p* < 0.05 among four groups). Besides, the most severe proteinuria was observed in the ELVFP group with 3.86 g/d (IQR: 1.00–7.01), followed by the LVH + ELVFP group with a level of 2.10 g/d (IQR: 0.61–4.68) (*p* < 0.05 among four groups). However, DSI showed no significant difference among the four groups (*p* > 0.05). Medications of RAASi and lipid-lowering drugs were found to be significantly different among the four groups (*p* < 0.05).

**Table 2:** Baseline characteristics stratified by cardiac function categories (*n* = 409).

Variables	Control patients (*n* = 195)	LVH (*n* = 116)	ELVFP (*n* = 34)	LVH + ELVFP (*n* = 64)	*p*-value
Age (years)	36.00 (29.00–50.00)	46.50 (36.25–55.00)^a^	53.00 (41.00–65.50)^a^	56.00 (47.25–65.00)^a^	*p* < 0.001
Gender (male/female)	135/60	21/95	28/6	21/43	*p* < 0.001
**Clinical parameter**					
BMI (kg/m^2^)	25.13 ± 3.78	23.43 ± 3.30^a^	25.77 ± 4.23	24.41 ± 3.61	*p* < 0.001
MAP (mmHg)	98.33 (88.33–105.33)	98.00 (88.17–106.00)	99.33 (87.25–111.00)	99.50 (90.58–111.75)	*p* = 0.227
**ECHO**					
LAD (mm)	32.00 (29.00–35.00)	32.00 (29.00–35.00)	35.00 (32.00–37.25)^a^	37.00 (32.00–40.00)^a^	*p* < 0.001
LVDd (mm)	45.00 (42.00–48.00)	46.00 (44.00–48.00)	47.00 (44.00–48.00)	48.00 (45.00–50.00)^a^	*p* < 0.001
LVMI (g/m^2^)	84.35 (73.42–92.97)	112.57 (103.55–123.22)^a^	89.08 (78.65–97.85)^b^	136.29 (114.57–149.78)^a^	*p* < 0.001
E/e’	6.40 (5.70–7.30)	7.00 (6.00–7.80)^a^	10.35 (9.50–11.30)^a^	10.70 (9.50–12.35)^a^	*p* < 0.001
LVEF (%)	64.40 (62.70–65.60)	64.00 (62.78–65.80)	64.00 (61.90–64.85)	64.40 (61.90–65.80)	*p* = 0.629
**Bioelectrical impedance**					
OH (L)	0.00(−0.70–0.90)	0.20(−0.70–1.17)	1.35 (0.43–4.35)^a^	1.05 (0.00–3.65)^a^	*p* < 0.001
TBW (L)	38.90 (33.40–44.80)	30.95 (27.53–35.23)^a^	41.05 (36.4–47.85)	33.45 (28.60–38.60)^a^	*p* < 0.001
ECW(L)	16.60 (14.30–19.10)	13.70 (12.30–15.68)^a^	19.55 (16.83–22.88)^a^	15.15 (13.73–19.08)	*p* < 0.001
ICW (L)	22.10 (18.50–25.20)	17.05 (15.10–20.00)^a^	20.95 (18.33–24.85)	17.55 (15.10–20.38)^a^	*p* < 0.001
ECW/ICW	0.76 (0.70–0.83)	0.79 (0.74–0.87)^a^	0.86 (0.76–1.02)^a^	0.87 (0.77–1.02)^a^	*p* < 0.001
OH/ECW (×10^−2^)	0.00(−4.65–4.82)	1.52(−5.57–8.43)	8.28 (2.17–20.48)^a^	6.94 (0.00–21.32)^a^	*p* < 0.001
ECW/TBW	0.432 (0.413–0.455)	0.441 (0.425–0.464)^a^	0.461 (0.433–0.505)^a^	0.466 (0.436–0.506)^a^	*p* < 0.001
**Laboratory parameter**					
Hb (g/L)	137.35 ± 19.26	124.95 ± 16.79^a^	132.00 ± 23.70	114.94 ± 21.58^a^	*p* < 0.001
ALb (g/L)	38.10 (33.20–41.00)	35.95 (28.48–39.20)^a^	29.55 (18.35–37.98)^a^	32.90 (25.23–37.60)^a^	*p* < 0.001
TC (mmol/L)	4.76 (4.06–5.94)	4.94 (4.15–6.26)	5.24 (4.45–7.65)	4.90 (4.39–6.21)	*p* = 0.266
TG(mmol/L)	1.47 (1.01–2.23)	1.50 (1.04–2.21)	1.81 (1.05–2.52)	1.43 (0.90–2.25)	*p* = 0.733
HDL-C (mmol/L)	1.09 (0.93–1.36)	1.24 (1.06–1.45)^a^	1.06 (0.93–1.45)	1.14 (0.96–1.46)	*p* = 0.010
LDL-C (mmol/L)	3.02 (2.42–3.62)	3.08 (2.37–3.87)	3.22 (2.62–4.16)	3.02 (2.69–3.62)	*p* = 0.759
eGFR (ml/min/1.73 m2)	98.01 (78.18–116.07)	90.88 (63.22–111.07)^b^	81.97 (53.06–106.14)^b^	67.12 (46.35–96.67)^a^	*p* < 0.001
CKD stage (1/2/3a/3b/4)	122/46/14/9/4	59/30/9/12/6	14/10/7/3/0	22/17/12/6/7	*p* < 0.001
Scr (umol/L)	80.00 (66.40–100.50)	68.85 (55.33–101.28)^b^	90.85 (64.78–125.93)	83.85 (62.85–115.90)	*p* = 0.017
24 h UP (g/d)	0.61 (0.25–1.96)	0.85 (0.20–2.53)	3.86 (1.00–7.01)^a^	2.10 (0.61–4.68)^a^	*p* < 0.001
24 h urinary sodium (mmol/d)	121.20 (86.40–171.00)	118.75 (80.15–163.53)	150.10 (104.53–192.10)	121.70 (87.90–144.83)	*p* = 0.068
DSI (g/d)	7.08 (5.05–9.99)	6.94 (4.68–9.55)	8.77 (6.11–11.22)	7.11 (5.14–8.46)	*p* = 0.068
**Medications**					
RAASi (%)	98 (50.25)	54 (46.55)	18 (52.94)	48 (75.00)	*p* = 0.002
Diuretic (%)	11 (5.64)	4 (3.45)	2 (5.88)	4 (6.25)	*p* = 0.762
Lipid-lowering drugs (%)	26 (13.33)	19 (16.38)	4 (11.76)	18 (28.13)	*p* = 0.040

BMI, body mass index; MAP, mean arterial pressure; ECHO, Echocardiography; LAD, left atrial dimension; LVDd, left ventricular end-diastolic dimension; LVMI, left ventricular mass index; LVEF, left ventricular ejection fraction; OH, overhydration; TBW, total body water; ECW, extracellular water; ICW, intracellular water; Hb, hemoglobin; ALb, albumin; TC, total cholesterol; TG, triglyceride; HDL-C, high-density lipoprotein cholesterol; LDL-C, low-density lipoprotein cholesterol; eGFR, estimated glomerular filtration rate; DSI, daily salt intake; RAASi, renin–angiotensin–aldosterone system inhibitors.

Data were presented as the mean ± SD, the median with interquartile range or counts, and percentages. A two-tailed *p* < 0.05 was considered statistically significant.

^a^Two-tailed *p* < 0.01(compared to the control patients); ^b^ Two-tailed *p* < 0.05(compared to the control patients).

### 3.3 Correlations between echocardiographic findings and basic parameters

**Figure 3:**
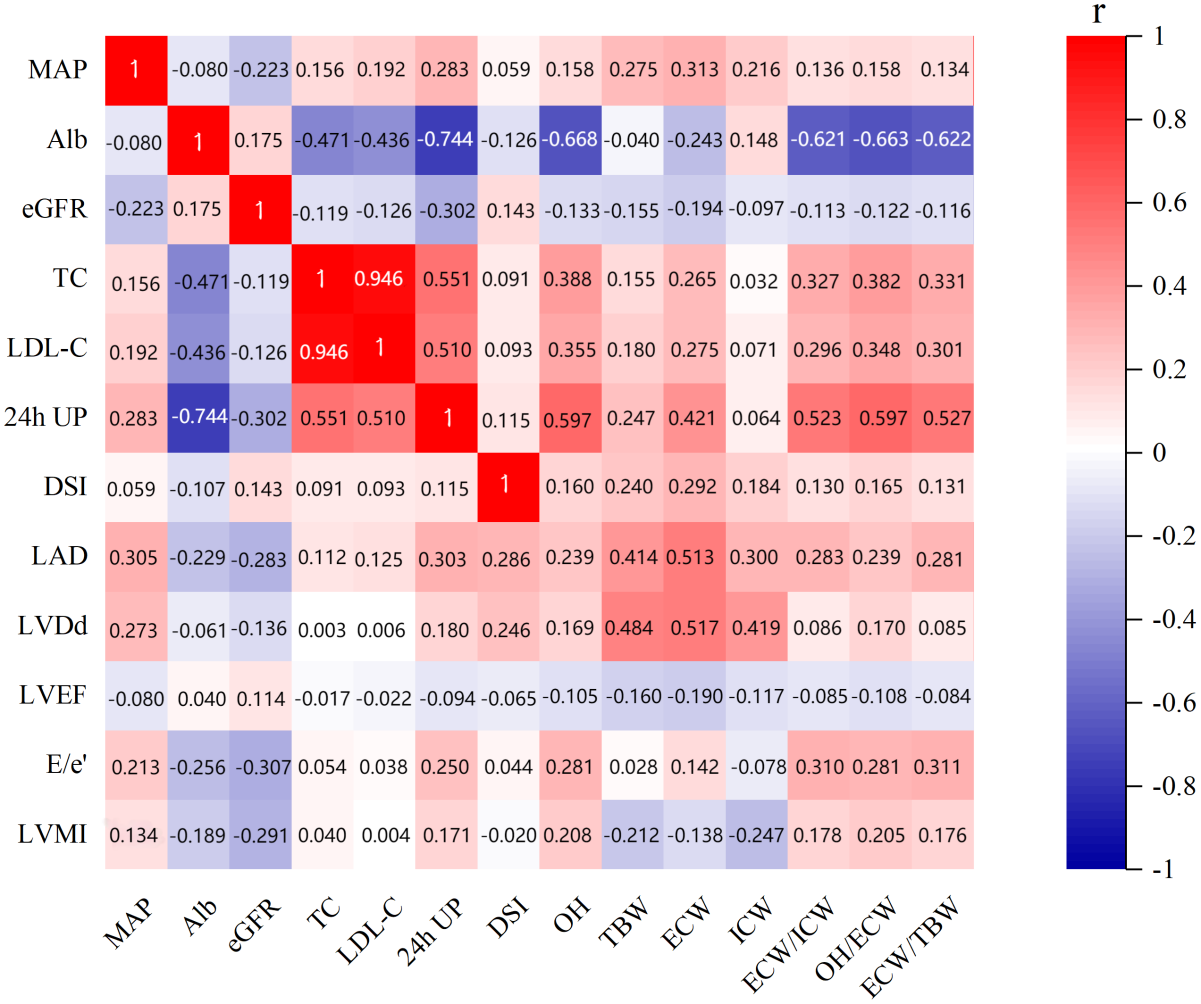
Correlations between echocardiographic parameters, body composition parameters, and clinical indicators. MAP, mean arterial pressure; LAD, left atrial dimension; LVDd, left ventricular end-diastolic dimension; LVMI, left ventricular mass index; LVEF, left ventricular ejection fraction; OH, overhydration; TBW, total body water; ECW, extracellular water; ICW, intracellular water; ALb, albumin; TC, total cholesterol; LDL-C, low-density lipoprotein cholesterol; eGFR, estimated glomerular filtration rate; DSI, daily salt intake; 24-h UP, 24-h urinary protein.

The correlations between echocardiographic parameters, body composition parameters, and clinical indicators were assessed using Spearman’s correlation analysis (Figure 3). ECW/TBW was found to be positively correlated with DSI (*r* = 0.131, *p* = 0.008); MAP (*r* = 0.134, *p* = 0.007); and lipid metabolic parameters including TC (*r* = 0.331, *p* < 0.001); LDL-C (*r* = 0.301, *p* < 0.001); but negatively correlated with renal function indicators including albumin (*r* = −0.622, *p* < 0.001) and eGFR (*r* = −0.116, *p* = 0.019). While DSI was positively correlated with eGFR (*r* = 0.143, *p* = 0.004), and fluid status indicators including OH (*r* = 0.160, *p* = 0.001); TBW (*r* = 0.240, *p* < 0.001); ECW (*r* = 0.292, *p* < 0.001); ICW (*r* = 0.184, *p* < 0.001); ECW/ICW (*r* = 0.130, *p* = 0.008); and OH/ECW (*r* = 0.165, *p* < 0.001); ECW/TBW (*r* = 0.131, *p* = 0.008), but negatively correlated with albumin (*r* = −0.107, *p* = 0.030).

Moreover, this showed that LVMI was negatively correlated with renal function parameters albumin (*r* = −0.189, *p* < 0.001) and eGFR (*r* = −0.291, *p* < 0.001); fluid status indicators including TBW (*r* = −0.212, *p* < 0.001); ECW (*r* = −0.138, *p* = 0.005); and ICW (*r* = −0.247, *p* < 0.001) and positively correlated with OH (*r* = 0.208, *p* < 0.001); ECW/ICW (*r* = 0.178, *p* < 0.001); OH/ECW (*r* = 0.205, *p* < 0.001); ECW/TBW (*r* = 0.176, *p* < 0.001); MAP (*r* = 0.134, *p* = 0.007) and 24-h UP (*r* = 0.171, *p* < 0.001). Meanwhile, E/e’ showed negative correlations with albumin (*r* = −0.256, *p* < 0.001) and eGFR (*r* = −0.307, *p* < 0.001), but positive correlations with MAP (*r* = 0.213, *p* < 0.001); OH (*r* = 0.281, *p* < 0.001); ECW (*r* = 0.142, *p* = 0.004); ECW/ICW (*r* = 0.310, *p* < 0.001); OH/ECW (*r* = 0.281, *p* < 0.001); ECW/TBW (*r* = 0.311, *p* < 0.001); and 24-h UP (*r* = 0.250, *p* < 0.001).

### 3.4 Association between DSI, ECW/TBW, and cardiac abnormalities

As shown in Table 3, the association between DSI and ECW/TBW among the study groups and the risks for the incidence of LVH or ELVFP was determined by binary logistic regression models. In the unadjusted model group, group 2 (low DSI & high ECW/TBW) and group 4 (high DSI & high ECW/TBW) had a 2.5-times and 2.217-times risk, respectively, for the diagnosis of LVH or ELVFP than group 1 (low DSI & low ECW/TBW) (group 2: OR = 2.500, 95%CI = 1.259–4.966, *p* = 0.009; group 4: OR = 2.217, 95%CI: 1.293–3.804, *p* = 0.004). After adjusting for age, sex, and BMI, the association between group 4 and an increased risk for the incidence of LVH or ELVFP was still significant with an OR of 2.351 (95%CI: 1.262–4.380, *p* = 0.007, model 1 in Table 3). This increased risk remained in group 4, even after extensive adjustment for model 1 plus MAP, albumin, eGFR, TC, and LDL-C (OR = 2.782, 95%CI = 1.427–5.426, *p* = 0.003, model 2 in Table 3). Additionally, the findings remained in a fully adjusted model including model 2 plus the use of RAASi, diuretics, and lipid-lowering drugs, in which group 4 was associated with a 2.396-times higher likelihood of LVH or ELVFP occurring, as compared to group 1 (OR = 2.396, 95%CI = 1.171–4.902, *p* = 0.017, model 3 in Table 3).

**Table 3:** Association between DSI and ECW/TBW in groups and cardiac function (*n* = 409).

Variables	Groups			
	Group 1:low DSI & low ECW/TBW	Group 2:low DSI & high ECW/TBW	Group 3:high DSI & low ECW/TBW	Group 4:high DSI & high ECW/TBW
No. (%)	88 (22.52%)	58 (14.18%)	118 (26.89%)	145 (35.45%)
Unadjusted ORs	Reference	2.500 (1.259–4.966)	0.871 (0.497–1.525)	2.217 (1.293–3.804)
*p*-value		*p* = 0.009	*p* = 0.629	*p* = 0.004
**Adjusted ORs adjusted for:**				
Model 1^a^	Reference	1.830 (0.843–3.973)	1.180 (0.635–2.191)	2.351 (1.262–4.380)
*p*-value		*p* = 0.126	*p* = 0.601	*p* = 0.007
Model 2^b^	Reference	1.692 (0.747–3.830)	1.558 (0.805–3.016)	2.782 (1.427–5.426)
*p*-value		*p* = 0.207	*p* = 0.188	*p* = 0.003
Model 3^c^	Reference	1.660 (0.703–3.921)	1.542 (0.792–3.000)	2.396 (1.171–4.902)
*p*-value		*p* = 0.248	*p* = 0.203	*p* = 0.017

BMI, body mass index; MAP, mean arterial pressure; ALb, albumin; eGFR, estimated glomerular filtration rate; TC, total cholesterol; LDL-C, low-density lipoprotein cholesterol; RAASi, renin–angiotensin–aldosterone system inhibitors.

^a^Model 1 adjusted for age, gender and BMI.

^b^Model 2 adjusted for age, gender, BMI, MAP, albumin, eGFR, TC, LDL-C.

^c^Model 3 adjusted for age, gender, BMI, MAP, albumin, eGFR, TC, LDL-C, use of RAASi, use of diuretic and use of Lipid-lowering drugs.

### 3.5 Prediction performance of DSI and ECW/TBW on cardiac abnormalities

**Figure 4:**
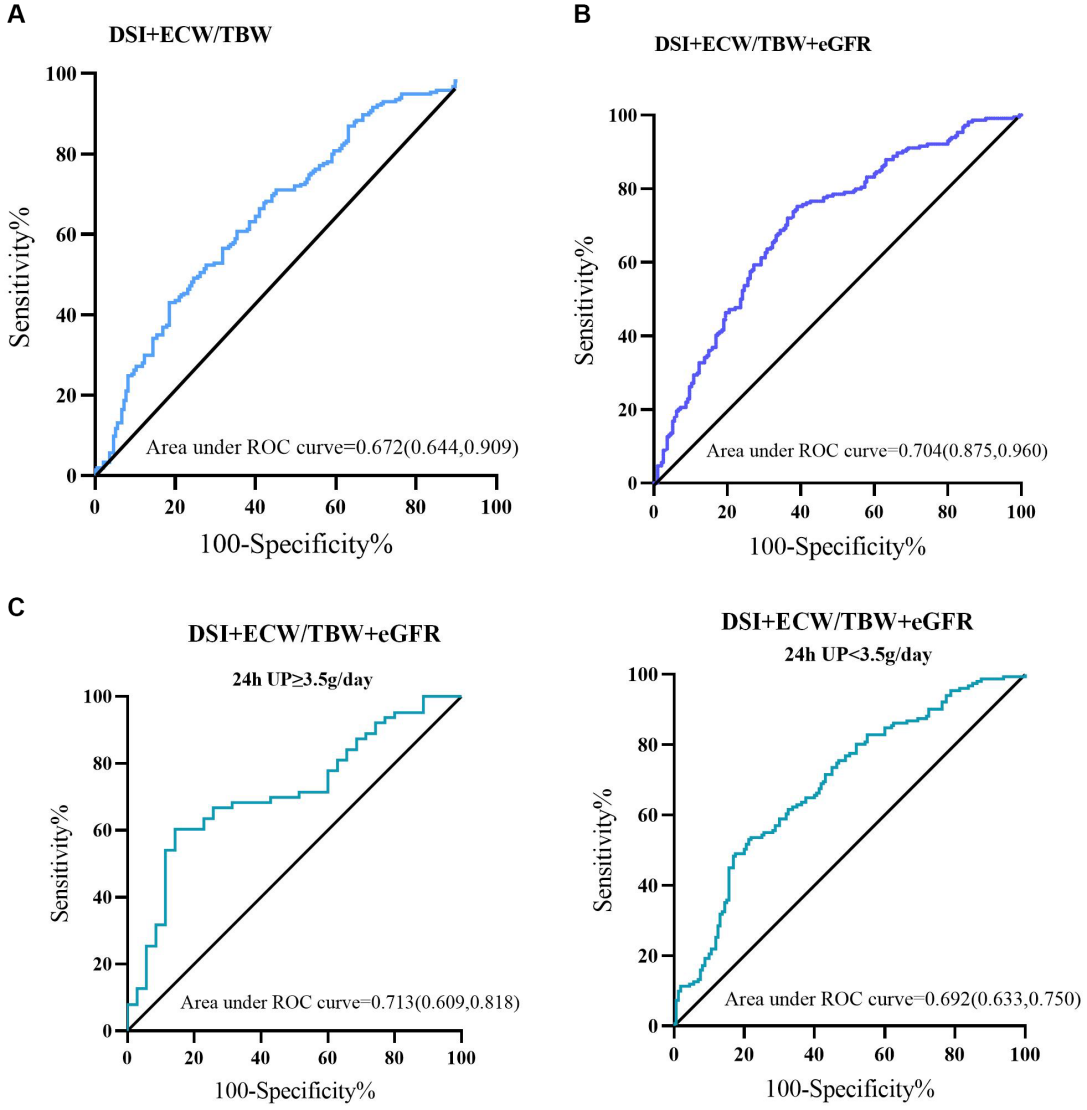
Predictive performances of DSI and ECW/TBW for the incidence of LVH or ELVFP evaluated by ROC curves. ROC curves of **(A)** DSI and ECW/TBW, **(B)** adding eGFR to DSI and ECW/TBW for the occurrence of LVH or ELVFP, and in those with nephrotic or non-nephrotic proteinuria **(C)**. AUC, area under the ROC curve; ROC, receiver operating characteristic; TBW, total body water; ECW, extracellular water; DSI, daily salt intake; LVH, left ventricular hypertrophy; ELVFP, elevated left ventricular filling pressure.

We used ROC analysis to evaluate the predictive value of DSI and ECW/TBW on the incidence of LVH or ELVFP. A combination of DSI and ECW/TBW showed an AUC of 0.673 (sensitivity = 71.0%, specificity = 54.9%) for predicting the occurrence of LVH or ELVFP. Moreover, adding DSI and ECW/TBW to eGFR boosted the predictive ability on the incidence of LVH or ELVFP with an AUC of 0.704 (sensitivity = 75.2%, specificity = 61.0%). According to different levels of 24-h UP (<3.5 or ≥ 3.5 g/day), the combination of DSI, ECW/TBW, and eGFR showed an AUC of 0.713 (sensitivity = 60.3%, specificity = 85.7%) for the incidence of LVH or ELVFP in patients with nephrotic proteinuria (Figure 4).

### 3.6 Subgroup analysis

To evaluate the modification effects of subgroups on the relationship between DSI, ECW/TBW, and cardiac dysfunction, we performed subgroup analyses in subgroups stratified by serum albumin (≤30 or > 30 g/L), eGFR (≤60 or > 60 mL/min/1.73 m^2^), BMI (≤25 kg/m^2^ or > 25 kg/m^2^), 24-h UP (<3.5 or ≥ 3.5 g/day), history of hypertension (yes or no), and history of DM (yes or no). Hig DSI and ECW/TBW (group 4) was strongly correlated with higher risks of the incidence of LVH or ELVFP (OR = 1.151, 95%CI = 1.003–1.321, *p* = 0.046). Moreover, the *p* values for interactions were > 0.05 for the subgroups by serum albumin, eGFR, BMI, 24-h UP, and history of hypertension and DM, suggesting that the increased risk of diastolic dysfunction associated with higher DSI and ECW/TBW was evident regardless of these factors (Table 4).

**Table 4:** Subgroup analysis.

Characteristics	Events, %	Group 1 OR (95%CI)	Group 2 OR (95%CI), *p*	Group 3 OR (95%CI), *p*	Group 4 OR (95%CI), *p*
All patients	214 (52.3)	1(Reference)^a^	1.092 (0.93, 1.282), 0.286^a^	1.069 (0.946, 1.208), 0.285^a^	1.151 (1.003, 1.321), 0.046^a^
**Serum albumin**					
>30 g/L	135 (63.1)	1(Reference)	1.038 (0.846, 1.273), 0.723	1.018 (0.892, 1.161), 0.796	1.123 (0.954, 1.322), 0.165
≤30 g/L	79 (36.9)	1(Reference)	1.46 (0.918, 2.322), 0.113	1.647 (0.995, 2.727), 0.055	1.485 (0.948, 2.324) 0.087
*p* for interaction		Reference	0.552	0.915	0.275
**eGFR**					
>60.ml.min.1.73m^2^	152 (71.0)	1(Reference)	1.094 (0.904, 1.324), 0.356	1.074 (0.937, 1.231), 0.304	1.129 (0.963, 1.324), 0.136
≤60.ml.min.1.73m^2^	62 (29.0)	1(Reference)	1.063 (0.755, 1.495), 0.729	1.031 (0.746, 1.424), 0.855	1.168 (0.855, 1.597), 0.332
*p* for interaction		Reference	0.213	0.903	0.503
**BMI**					
≤25 kg/m2	129 (60.3)	1(Reference)	1.137 (0.925, 1.398), 0.224	1.143 (0.979, 1.335), 0.092	1.187 (0.98, 1.437), 0.081
>25 kg/m2	85 (39.7)	1(Reference)	1.065 (0.806, 1.408), 0.657	0.927 (0.747, 1.15), 0.49	1.073 (0.861, 1.337), 0.53
*p* for interaction		Reference	0.162	0.073	0.478
**Proteinuria**					
<3.5 g/d	151 (70.6)	1(Reference)	1.083 (0.895, 1.312), 0.412	1.061 (0.931, 1.209), 0.373	1.147 (0.981, 1.34), 0.087
≥3.5 g/d	63 (29.4)	1(Reference)	1.181 (0.698, 1.998), 0.537	1.104 (0.637, 1.912), 0.725	1.174 (0.71, 1.942), 0.533
*p* for interaction		Reference	0.724	0.935	0.789
**History of hypertension**					
No	126 (58.9)	1	1.405 (0.342, 5.997), 0.639	1.625 (0.503, 5.375), 0419	1.349 (0.41, 4.406), 0.619
Yes	88 (41.1)	1	1.603 (0.482, 5.387), 0.44	1.181 (0.486, 2.927), 0.715	2.715 (0.962, 7.959), 0.063
*p* for interaction			1	0.017	0.987
**History of DM**					
No	171 (79.9)	1	1.823 (0.706, 4.833), 0.219	1.415 (0.708, 2.867), 0.33	2.136 (0.958, 4.829), 0.065
Yes	43 (20.1)	1	1.184 (0.034, 47.386), 0.925	5.586 (0.227, 207.392),0.31	2.557 (0.121, 59.439), 0.541
*p* for interaction			0.718	0.965	0.846

^a^Adjusted for age, gender, BMI, MAP, albumin, Hb, eGFR, TC, LDL-C, use of ACEI/ARB, use of diuretic and use of Lipid-lowering drugs.

BMI, body mass index; MAP, mean arterial pressure; ECHO, Echocardiography; Hb, hemoglobin; ALb, albumin; TC, total cholesterol; TG, triglyceride; HDL-C, high-density lipoprotein cholesterol; LDL-C, low-density lipoprotein cholesterol; eGFR, estimated glomerular filtration rate; DM, diabetes mellitus.

## 4 Discussion

The present study strengthens the association between a combination of sodium excretion and volume overload and risks of cardiac structural and functional impairments in a population with non-dialysis CKD. These analyses document a significantly increased risk of LVH or ELVFP occurrence in individuals with both high DSI (≥6 g/d) and high ECW/TBW (≥median value 0.439), independent of important CKD and CVD risk factors. Compared to group 1 (low DSI and low ECW/TBW), group 4 (high DSI and high ECW/TBW) showed a 2.396-fold (95%CI: 1.171–4.902; *p* = 0.017) high risk of LVH or ELVFP incidence in a fully adjusted model. Furthermore, ROC analysis confirmed the predictive ability with an AUC of 0.673 with a high sensitivity but only fair specificity. More importantly, combined with eGFR, DSI and ECW/TBW could identify patients with higher cardiac dysfunction risk estimates with a satisfactory accuracy (AUC = 0.704, sensitivity = 75.2%, specificity = 61.0%). The specificity was improved to 85.7% in those with nephrotic proteinuria (AUC = 0.713). These findings were consistent across subgroups that strongly supported the impact of the combination of DSI and ECW/TBW on cardiac structural and functional impairments in CKD patients.

The ECW/TBW ratio which is considered an “edema index,” served as a significant predictor of poorer outcomes indicating a composite of overhydration and protein-energy wasting (31, 34). It evaluated extracellular fluid (ECF) status by body composition monitor based on cell membrane resistance to frequency currents (35). ECF excess is a proven common characteristic of advanced ongoing-dialysis CKD and has a close association with all-cause mortality and CV morbidity (34–36). Renal function loss resulted in reduced excretion of sodium and water, thereby resulting in ECF excess (37). Consistently, our results revealed that ECW/TBW was negatively correlated with eGFR. Moreover, ECW/TBW was found to be significantly correlated with conventional CV risk factors as indicated by MAP, TC, and LDL-C values, which further support that ECF excess contributed to hypertension, HF, and pulmonary vascular congestion in CKD patients (37, 38). Previous studies have shown that in the presence of ECF excess, urinary excretion of sodium and fluid was increased through a negative feedback mechanism to maintain fluid homeostasis. Disruption of water and salt homeostasis and a reduction in the ability to perform natriuresis were classic features and the major underlying pathogenesis of CKD that are often coupled with the expansion of the extracellular water (ECW) compartment (39). Accordingly, ECW/TBW was positively correlated with DSI in our study. Our study added evidence to the potential relationship of ECW/TBW and DSI in different CKD stages. As has been suggested in previous literatures, when renal function deteriorates, urinary excretion of sodium and fluid is reduced along with triggering of the intrarenal RAAS, leading to persistent and exacerbated ECF excess (34, 40).

Accumulating evidence indicated that high intake of dietary salt was an established risk factor for not only renal function loss but also CVD incidence in CKD (19, 41). Owing to impaired tubuloglomerular feedback and increased renal sodium reabsorption, blood pressure was even more sensitive to high sodium intake in CKD (42). One prospective cohort study of CKD patients from the Chronic Renal Insufficiency Cohort Study suggested that higher urinary sodium excretion was associated with increased risk of CVD (17). Another study revealed that 24-h urinary sodium excretion was associated with CKD progression and all-cause mortality among 3,757 patients with CKD in the Chronic Renal Insufficiency Cohort Study (43). In addition, higher DSI (>6 g/day) was significantly associated with renal outcome than lower DSI (≤6 g/day), and strict salt restriction has reportedly shown an association with improvements in hypertension and urinary protein reduction overload (44, 45). However, the association of salt intake and CKD progression is still controversial. Fan et al. found that when DSI was under 3 g/day, higher urinary sodium was associated with increased risk of kidney failure in those with baseline proteinuria under 1 g/day and with lower risk of kidney failure than in those with baseline proteinuria ≥1 g/day (46). Another study revealed that in patients with advanced CKD (eGFR<30 mL/min/1.73 m^2^), sodium intake does not impact the CKD progression (47). In addition, low sodium intake was also associated with kidney failure in those with macroalbuminuria (48). In the current study, DSI was positively correlated with eGFR. Patients in group 2 (low DSI and high ECW/TBW) but not in group 4 (high DSI and high ECW/TBW) had the most severe renal function and the highest ratio of CKD stages G3 and G4. S A possible explanation for the discrepancy was the inaccuracy of the 24-h urine sample collections and small sample sizes. Combined with our results, low DSI may be because of impaired renal function in advanced CKD, resulting in reduced urine volume and decreased urinary sodium excretion. Taken together, it may be more appropriate to take the CKD stages into account when analyzing the effect of DSI on CVD in CKD patients. The addition of eGFR to DSI and ECW/TBW which resulted in improvements of predictive performance of LVH or ELVFP incidence supported this hypothesis.

LVH was not only the most frequent structural cardiac abnormality in patients with CKD but also an important risk factor for adverse CV outcomes in patients with CKD (21, 49). Cerasola et al. reported a progressive increase of LVH prevalence with decreasing renal function and an inverse association between GFR and LVM, independent of potential confounders (50). Additionally, several studies suggested that the risk of LVH was significantly associated with CKD severity, with increasing prevalence correlating with a deterioration in renal function (4, 13). Besides, high albumin excretion was independently related to LVH (51). Consistent with previous studies, our results indicated that LVMI was negatively correlated with albumin and eGFR, but positively correlated with MAP and 24-h UP, which reiterated the necessity and importance of early detection of LVH and LV dysfunction in CKD patients. Furthermore, diastolic LV filling is complex and hence, the echocardiographic assessment of the value of E/e’ was a robust marker of LVFP and helpful to diagnose LV diastolic dysfunction (7, 15). Elevated E/e’ ratio has been reported to be associated with increased mortality in a range of CVDs including HF with reduced EF, mitral and aortic regurgitation, aortic stenosis, and hypertension (7, 15). E/e’ remained a marker of increased mortality with a pivot point of increased mortality at ~9, which was also the diagnostic cut-off value to define ELVFP (7, 10, 12). In the current study, E/e’ showed significant correlations with renal function and proteinuria. In our study, those in the ELVFP group had the lowest eGFR and highest level of 24-h UP, further indicating the association between CKD and increased risk of CV morbidity and mortality. Therefore, the identification of novel risk factors in combination with traditional risk factors might enable extensive documentation and analysis of patients at high risk of CVD incidence, thereby preventing CV diseases in CKD patients in an opportune and efficient manner to improve survival.

The primary finding of the study was an association of the combination of higher DSI and ECW/TBW with an increased risk of incidence of LVH or ELVFP in CKD, highlighting the critical role of sodium and fluid overload in CV morbidity future mortality in CKD patients. A high-salt diet could induce sodium retention in CKD patients, leading to fluid overload which, in turn, can cause pressure overload contributing to CVD risk (44, 45). Direct effects of DSI on RAAS activity were also suggested in the association of DSI and CVD (52). Moreover, the findings reported here are independent of adjustment for conventional important CVD risk factors such as MAP; albumin; eGFR; TC; and LDL-C and the use of RAASi, diuretics, and lipid-lowering drugs, suggesting that other potential mechanisms likely play a role in the effect of sodium and fluid overload on CVD in CKD patients. Hung et al. suggested that fluid retention in patients or animals was associated with renal inflammation with macrophage infiltration and tumor necrosis factor-α overexpression, glomerular sclerosis, and cardiac fibrosis (21). The other potential mechanisms involved in the direct effects of DSI on CVD included endothelial dysfunction, increased oxidative stress leading to vascular damage, and insulin resistance (17). In addition, several research data have shown that CKD is involved in many pathophysiological pathways that potentially contribute to the elevated risk of CVD observed in patients with CKD, and the relative contributions of these mechanisms varied across CKD severity (14). Our findings confirmed these observations that DSI and ECW/TBW as non-traditional risk factors which were also related to CKD progression, broadened group of CV risk factors and the prevalence increases with declining eGFR. More importantly, although the effect of DSI and ECW/TBW on CVD was independent of adjusting eGFR, adding DSI and ECW/TBW to eGFR could greatly boost the prediction ability of LVH or ELVFP incidence in CKD patients. They showed high specificity especially in those with nephrotic proteinuria. These results not only reflected a shared sodium and fluid overload linking CKD to CVD but also suggested the need for timely targeting of the risk factors on the CV system even in the early stages of the disease.

### 4.1 Limitations

This study has some limitations. First, as a cross-sectional observational analysis, this study could not establish the causality of the relationship between sodium and volume overload and outcomes. Second, DSI and ECW/TBW were measured at enrollment and not serially. Thus, the results of single-point measurements may not clearly reflect the impact of fluid and sodium changes over time or the time-averaged exposure on CVD incidence. Third, it lacked follow-up data concerning future CV events which limits the study’s predictive validity. Hence, further large, well-designed, prospective studies with multiple-measurements during follow-up are warranted to ascertain the short- and long-term efficacy of DSI and ECW/TBW on CVD in CKD patients.

## 5 Conclusion

We translate salt and volume status into clinical practice. The combination of high DSI (>6 g/d) and high ECW/TBW (>0.439) was associated with a greater risk of LVH or ELVFP incidence in non-dialysis CKD patients, independent of age, sex, BMI, MAP, albumin, eGFR, TC, LDL-C and the use of RAASi, diuretics, and lipid-lowering drugs. Moreover, adding well-accepted and widely-used parameters eGFR and proteinuria into consideration improved the diagnostic ability of DSI and ECW/TBW for risk stratification of cardiac impairments in CKD patients.

Our findings suggest that the salt and volume overload may be important mechanisms contributing to the CVD morbidity and mortality observed in non-dialysis CKD. Moreover, the findings establish salt and volume status reference ranges for risk stratification of future CVD in CKD. Timely modification of the salt and fluid status in non-dialysis CKD patients may be beneficial to achieve better CVD outcomes and therefore decrease the burden of mortality.

## Data availability statement

The raw data supporting the conclusions of this article will be made available by the authors, without undue reservation.

## Ethics statement

The studies involving humans were approved by Ethics Committee of the First Affiliated Hospital of Nanjing Medical University. The studies were conducted in accordance with the local legislation and institutional requirements. Written informed consent for participation in this study was provided by the participants or his/her legal guardians/next of kin.

## Author contributions

SD: Writing – review & editing, Writing – original draft, Conceptualization. YM: Writing – original draft, Formal analysis, Data curation. FL: Writing – review & editing, Software, Formal analysis. CZ: Writing – original draft, Software, Investigation. HG: Writing – review & editing, Methodology, Data curation. MZ: Writing – review & editing, Data curation. BS: Writing – review & editing, Data curation. YY: Writing – review & editing, Data curation. CX: Writing – review & editing. HM: Writing – review & editing, Supervision. BZ: Validation, Writing – review & editing, Writing – original draft, Supervision, Funding acquisition.
